# Gamma knife radiosurgery versus transcatheter arterial chemoembolization for hepatocellular carcinoma with portal vein tumor thrombus: a propensity score matching study

**DOI:** 10.1007/s12072-022-10339-2

**Published:** 2022-06-21

**Authors:** Ke Su, Tao Gu, Ke Xu, Jing Wang, Hongfei Liao, Xueting Li, Lianbin Wen, Yanqiong Song, Jiaqi Zhong, Bingsheng He, Xin Liu, Jie He, Yanlin Liu, Qi Li, Xunjie Feng, Siyu Chen, Binbin Yang, Weihong Huang, Hongping Jin, Xiaotong Luo, Teng Hu, Jiali Chen, Zhenying Wu, Simin Lu, Jianwen Zhang, Mingyue Rao, Yunchuan Xie, Jing Wang, Xiaoning Zhu, Lan Chen, Bo Li, Song Su, Xiaoli Yang, Juan Wang, Hao Zeng, Pan Wang, Min Yan, Xiaojing Chen, Kun He, Yunwei Han

**Affiliations:** 1 Department of Oncology, The Affiliated Hospital of Southwest Medical University, Luzhou, 646000 China; 2 Clinical Research Institute, The Affiliated Hospital of Southwest Medical University, Luzhou, 646000 China; 3 Department of Body Gamma Knife Treatment Room, 363 Hospital, Chengdu, 610041 China; 4 Department of Oncology, 363 Hospital, Chengdu, 610041 China; 5 Department of Geriatric Cardiology, Sichuan Academy of Medical Sciences & Sichuan Provincial People’s Hospital, Chengdu, 610072 China; 6 Department of Radiotherapy, Sichuan Cancer Hospital & Institute, Sichuan Cancer Center, School of Medicine, University of Electronic Science and Technology of China, Chengdu, 610042 China; 7 Clinical Medical College, Southwest Medical University, Luzhou, 646000 China; 8 Department of Radiology, The Affiliated Hospital of Southwest Medical University, Luzhou, 646000 China; 9 Department of Hepatobiliary, The Affiliated Traditional Chinese Medicine Hospital of Southwest Medical University, Luzhou, 646000 China; 10 Department of Oncology and Hematology, The Affiliated Traditional Chinese Medicine Hospital of Southwest Medical University, Luzhou, 646000 China; 11 Department of General Surgery (Hepatobiliary Surgery), The Affiliated Hospital of Southwest Medical University, Luzhou, 646000 China; 12 Clinical Skills Center, The Affiliated Hospital of Southwest Medical University, Luzhou, 646000 China; 13 Nuclear Medicine and Molecular Imaging Key Laboratory of Sichuan Province, Luzhou, 646000 China; 14 Academician (Expert) Workstation of Sichuan Province, Luzhou, 646000 China; 15 School of Basic Medical Sciences, Shandong University, Jinan, 250011 China

**Keywords:** Gamma knife radiosurgery, Radiotherapy, Transcatheter arterial chemoembolization, Hepatocellular carcinoma, Primary liver cancer, Portal vein tumor thrombus, Propensity score matching, Overall survival, Cheng’s classification, Advanced stage

## Abstract

**Background:**

The optimal locoregional treatment for hepatocellular carcinoma (HCC) patients with portal vein tumor thrombus (PVTT) is unclear. This study aimed to investigate the efficacy of Gamma knife radiosurgery (GKR) versus transcatheter arterial chemoembolization (TACE) in HCC patients with PVTT.

**Methods:**

This retrospective study included 544 HCC patients with PVTT (GKR, 202; TACE, 342). Propensity score matching (PSM) analysis identified 171 matched pairs of patients. The primary endpoint was overall survival (OS).

**Results:**

Before PSM, the GKR group exhibited longer median OS (mOS) than the TACE group (17.2 vs. 8.0 months, *p* < 0.001). We followed the Cheng’s classification for PVTT. In the subgroup analysis, GKR was associated with significantly longer mOS for patients with PVTT II-IV (17.5 vs. 8.7 months, *p* < 0.001; 17.2 vs. 7.8 months, *p* = 0.001; 14.5 vs. 6.5 months, *p* = 0.001, respectively) and comparable OS for patients with PVTT I. After PSM, the GKR group had also a longer mOS than the TACE group (15.8 vs. 10.4 months, *p* < 0.001). In the subgroup analysis, the GKR group demonstrated superior mOS for patients with PVTT II-IV (all *p* < 0.05) and comparable OS for patients with PVTT I.

**Conclusions:**

GKR was associated better OS than TACE in HCC patients with PVTT, especially for patients with PVTT II-IV.

**Clinical Trials Registration:**

The study was registered in the Chinese Clinical Trials Registry under the registration number ChiCTR2100051057.

**Supplementary Information:**

The online version contains supplementary material available at 10.1007/s12072-022-10339-2.

## Introduction

Hepatocellular carcinoma (HCC) is characterized by high morbidity and death [1]. HCC is prone to invade the portal vein system and can cause portal vein tumor thrombus (PVTT), which is a poor prognostic factor and results in a median overall survival (mOS) of only 2–4 months [2–4]. The Barcelona Clinic Liver Cancer staging system classified patients with PVTT to be at an advanced stage and recommended atezolizumab plus bevacizumab as the first-line treatment [5]. In addition, recently presented results of durvalumab plus tremelimumab might add another option [6]. However, the objective response rate (ORR) of unresectable HCC patients receiving atezolizumab plus bevacizumab was only 27.3%. It is critical to explore other therapeutic options to improve local control of advanced HCC patients.

According to current literature, liver resection (LR) is the preferred treatment in PVTT I/II patients with Child–Pugh A and Eastern Cooperative Oncology Group performance status (ECOG PS) 0–1 [7, 8]. However, for patients with other types of PVTT, there is still controversy regarding the best locoregional treatment [9].

With the advancement of local therapeutic techniques, extremely TACE, radiofrequency ablation (RFA), radiotherapy including gamma knife radiosurgery (GKR) and stereotactic body radiation therapy (SBRT) have become feasible. HCC patients with PVTT receiving TACE extended months mOS to 4–10 months [10, 11]. But as an invasive treatment, TACE has a great impact on the life of the patient and carries risks such as liver failure. GKR is a form of external radiotherapy and can allow delivery of ablative doses of radiation with low toxicity. A retrospective study of small sample of patients reported that the mOS of HCC patients with PVTT who received GKR was 6.1 months (95%CI: 4.7–7.5) [12]. In addition, a study by Xiaojie and colleagues reported that patients with HCC-derived PVTT who received GKR combined with TACE had longer OS than those treated with TACE alone [13]. GKR and TACE had shown strong therapeutic prospect for HCC with PVTT.

Although HCC patients with PVTT who do not undergo surgery are candidates for both GKR and TACE, to the best of our knowledge, no data comparing these modalities is available. Therefore, we conducted this study to compare the effectiveness of GKR versus TACE for HCC patients with PVTT.

## Materials and methods

### Patients

This is a multicentre, retrospective, cohort analysis of HCC patients with PVTT treated with GKR or TACE between June, 2015 and July, 2021 at three Chinese tertiary hospitals.

GKR was recommended in HCC patients with PVTT who had Child–Pugh A/B, an ECOG PS of 0–1, a minimum of 700 mL of uninvolved liver, no obstinate ascites or hepatic encephalopathy, and adequate organ function defined as creatinine ≥ 1.5 × upper limit of normal (ULN), absolute neutrophil counts (ANC) ≥ 1.5 × 109/l, international normalised ratio (INR) < 1.7, alanine transaminase (ALT) or aspartate transaminase (AST) < 2.5 × ULN.

Patients with diffusely infiltrative disease or more than five tumor nodules were not recommended for GKR.

TACE was recommended in HCC patients with PVTT who had Child–Pugh A/B, no obstinate ascites or hepatic encephalopathy, and an ECOG PS of 0–2. Patients with Child–Pugh C, inadequate organ function, or an ECOG PS of 3 or 4 were not recommended for TACE.

The exclusion criteria were as follows: (1) patients concomitantly received other locoregional treatments such as TACE plus GKR, hepatic resection, or radiofrequency ablation; (2) patients with obstinate ascites, hepatic encephalopathy; (3) patients with cancers other than HCC; (4) inadequate clinical data; (5) patients who had used any locoregional treatment within 12 weeks prior to the first treatment in this study; (6) diffusely infiltrative disease or more than five tumor nodules; (7) Child–Pugh C, inadequate organ function, residual liver volume < 700 ml, or an ECOG PS ≥ 2; (8) both GKR and TACE cannot be adequately performed.

Physicians explained the two treatment options to all eligible patients. A final treatment decision was determined by physicians after discussion with the Hospital HCC Expert Team including hepatologists, radiation oncologists, surgeons, interventional radiologists, medical oncologists, and pathologists, and taking into account the physician’s and patient’s preferences, and treatment costs. All patients signed informed consents before they received any treatment.

Before patients received GKR or TACE, sorafenib was recommended by the physicians. If the patient agreed to the recommendation, sorafenib was administered after the first GKR or TACE session. Patients who refused sorafenib underwent GKR or TACE only.

### Classification of PVTT

We followed the Cheng’s classification for PVTT and classified the types of PVTT according to four levels: (a) type I was defined as PVTT in the segmental or sectoral branches of the portal vein or above; (b) type II was defined as PVTT in the right/left portal vein; (c) type III was defined as PVTT in the main portal vein; and (d) type IV was defined as PVTT in the superior mesenteric vein. Type I0 represented tumor thrombus found only under microscopy observation [14–16].

### Treatment protocol

#### GKR

This is a multicenter study from three different centers with different numbers of radiation oncologists. When a patient accepted to receive GKR, the specific target dose was discussed by the center's radiation oncologists (2 or 3). The principal investigator (PI) of radiation oncology made the final decision. GKR was performed using Treatment Planning System (TPS) by the radiation oncologists who delineated the irradiation area as per contrast-enhanced CT scan. Delineation of the gross target volume (GTV) including the primary liver tumor and tumor thrombosis was done based on the image technology. The radiation oncologists defined a 5–10 mm margin around the GTV as the planning target volume (PTV) by TPS. The median tumor margin dose was 42 Gy (range 39–42 Gy), with a median isodose line of 50% (range 50–60%). Dose-volume histograms were used to protect adjacent normal tissues and the liver, as delineated during the target planning process. The course of GKR treatment was divided into 2 cycles with 7 times per course (once a day). The radiation oncologists revised the treatment plan by CT after the first course of treatment, and ahead of the second course of treatment. A second GKR plan was made based on the delineations of the intra-treatment CT images. There was no interval between the 2 cycles.

#### TACE

TACE was performed by delivering an emulsion of lipiodol (10–20 ml) and one or more chemotherapeutic agents, such as cisplatin, or cisplatin and mitomycin-C, or Fluorouracil. The delivery was performed into the selected vessels after a microcatheter was advanced into the hepatic arterial circulation to the most distal tumor-feeding vessel, followed by embolization using gelatin sponge particles or other embolic materials if deemed necessary.

Repeated TACE procedures were performed in the case of multiple lesions or large lesions. The decision to perform multiple TACE was made by HCC Expert Team.

#### Patient follow-up and data collection

Post-treatment follow-ups were conducted every 2–3 months and entailed comparison of CT, or MRI and laboratory tests at baseline. Laboratory tests included biochemical and hematologic analyses, such as pro-thrombin time; complete blood cell count; and measurements of a-fetoprotein (AFP), total bilirubin, alkaline phosphatase (ALP), alanine aminotransferase (ALT), and aspartate aminotransferase (AST) levels.

### Statistical analysis

Categorical variables were analyzed using *χ*2 and McNemar analysis. One-to-one propensity score matching (PSM) was adopted to reduce selection bias and confounding factors resulting from different co-variable distribution among GKR and TACE groups. Independent variables included in the propensity model were sex, age, Child–Pugh classification, tumor number, tumor size, AFP levels, ALP levels, platelet levels, ALT levels, leukocyte levels, type of PVTT, HBV infection, HCV infection, drinking history, lymph node metastasis, extrahepatic metastasis, and previous therapy status. Survival was computed as the interval between the date of GKR or TACE and the date of death for any reason, with censoring at the date of last follow-up in surviving patients. Kaplan–Meier method with log-rank test was utilized to compare long-term survival distribution. In exploratory subgroup analyses, the OS of GKR and TACE in predefined subgroups was assessed using Cox proportional hazard models presented in a forest plot; forest plots show factors associated with OS. Univariate and multivariable Cox analyses of all data were done to confirm significant predictors for OS. Factors with *p* values less than 0.05 in univariate analysis were introduced into the multivariate Cox proportional hazards model to determine the adjusted hazard ratios (HRs) and 95% confidence intervals (CIs). All statistical analyses were conducted with SPSS for Windows (version 26.0). Two tailed *p* value < 0.05 were considered as statistically significant.

## Results

### Patient characteristics of the pooled and matched cohorts

Between June, 2015 and July, 2021, 4563 patients were diagnosed with HCC. Among them, 4019 patients did not meet the eligibility criteria and were excluded. We included 202 patients who received GKR and 342 patients who received TACE (Supplementary Fig. 1). Before matching, the median follow-up period was 25.0 months for the GKR group and 20.8 months for the TACE group. Patients who underwent TACE had more multiple tumors (*p* = 0.022), larger tumors (*p* = 0.011), higher AFP levels (*p* = 0.010), more lymph node metastases (*p* = 0.012), worse PVTT type (*p* = 0.035) than did those patients who underwent GKR. Patients in the GKR group had experienced a higher number of previous therapies (*p* < 0.001) than patients in the TACE group (Table 1).

**Table 1:** Baseline characteristics of the patients before and after PSM

Variable	Before PSM				After PSM		
	GKR	TACE	*p*		GKR	TACE	*P*
Patients	202	342			171	171	
Male sex	178 (88.1)	302 (88.3)	0.948		151 (88.3)	156 (91.2)	0.473
Age ≥ 60 years	75 (37.1)	106 (31)	0.142		57 (33.3)	63 (36.8)	0.561
Child–Pugh score			0.310				0.806
5	67 (33.2)	129 (37.7)			62 (36.3)	62 (36.3)	
6	76 (37.6)	108 (31.6)			57 (33.3)	55 (32.2)	
7	36 (17.8)	63 (18.4)			29 (17)	31 (18.1)	
8	10 (5)	27 (7.9)			10 (5.8)	14 (8.2)	
9	13 (6.4)	15 (4.4)			13 (7.6)	9 (5.3)	
Number of tumors ≥ 2	150 (74.3)	282 (82.5)	0.022		132 (77.2)	135 (78.9)	0.771
Tumor diameter, cm			0.011				0.182
<2	2 (1)	8 (2.3)			2 (1.2)	2 (1.2)	
≥2, <5	52 (25.7)	53 (15.5)			37 (21.6)	27 (15.8)	
≥5, <10	79 (39.1)	131 (38.3)			73 (42.7)	66 (38.6)	
≥10	69 (34.2)	150 (43.9)			59 (34.5)	76 (44.4)	
Serum AFP, ng/ml			0.010				0.495
<200	91 (45)	117 (34.2)			71 (41.5)	71 (41.5)	
≥200, <400	17 (8.4)	20 (5.8)			16 (9.4)	9 (5.3)	
≥400	94 (46.5)	205 (59.9)			84 (49.1)	91 (53.2)	
ALP levels ≥ 125 U/L	132 (65.3)	250 (73.1)	0.056		114 (66.7)	120 (70.2)	0.567
Platelet count ≥ 100 × 109/L	150 (74.3)	251 (73.4)	0.825		127 (74.3)	119 (69.6)	0.382
ALT levels ≥ 40 U/L	121 (59.9)	213 (62.3)	0.582		102 (59.6)	101 (59.1)	1.000
leukocyte ≥ 4 × 10^9^/L	159 (78.7)	272 (79.5)	0.820		139 (81.3)	134 (78.4)	0.590
Number of TACE ≥ 2	-	74 (21.6)			-	38 (22.2)	
Cheng’s type of PVTT			0.035				0.295
I	42 (20.8)	42 (12.3)			39 (22.8)	25 (14.6)	
II	62 (30.7)	134 (39.2)			56 (32.7)	74 (43.3)	
III	65 (32.2)	113 (33)			49 (28.7)	49 (28.7)	
IV	33 (16.3)	53 (15.5)			27 (15.8)	23 (13.5)	
Etiology							
HBV	134 (66.3)	208 (60.8)	0.198		113 (66.1)	108 (63.2)	0.672
HCV	4 (2)	9 (2.6)	0.631		3 (1.8)	4 (2.3)	1.000
Alcohol	85 (42.1)	138 (40.4)	0.692		73 (42.7)	65 (38)	0.451
Lymph node metastasis	100 (49.5)	207 (60.5)	0.012		89 (52)	93 (54.4)	0.731
Extrahepatic metastases	57 (28.2)	94 (27.5)	0.854		49 (28.7)	52 (30.4)	0.815
Lung	28 (13.9)	69 (20.2)			24 (14)	39 (22.8)	
Bone	24 (11.9)	15 (4.4)			19 (11.1)	8 (4.7)	
Other	19 (9.4)	23 (6.7)			18 (10.5)	14 (8.2)	
Combined sorafenib	53 (26.2)	95 (27.8)	0.697		68 (39.8)	66 (38.6)	0.078
Previous therapy	99 (49.0)	66 (19.3)	<0.001		99 (49.0)	66 (38.6)	0.791
Systemic therapy	43 (21.3)	8 (2.3)			30 (17.5)	8 (4.7)	
Liver resection	43 (21.3)	22 (6.4)			29 (17)	22 (12.9)	
Radiotherapy	18 (8.9)	0			13 (7.6)	0	
TACE	49 (24.3)	44 (12.9)			37 (21.6)	44 (25.7)	
RFA	14 (6.9)	4 (1.2)			11 (6.4)	4 (2.3)	

*PSM* propensity score matching, *AFP* alpha fetoprotein, *ALP* alkaline phosphatase, *ALT* alanine aminotransferase, *PVTT* portal vein tumor thrombus, *HBV* hepatitis B virus, *HCV* hepatitis C virus, *GKR* gamma knife radiosurgery, *TACE* transcatheter arterial chemoembolization, *RFA* radiofrequency ablation

After performing PSM, we identified 171 matched pairs of patients with comparable patient and tumor characteristics. In this matched cohort, patients in the GKR and TACE groups were not significantly different with regard to any patient or tumor covariates at baseline (Table 1). The median follow-up time was 25.5 months in the GKR group and 19.8 months in the TACE group.

### Overall survival analyses in the pooled and matched cohorts

**Fig. 1:**
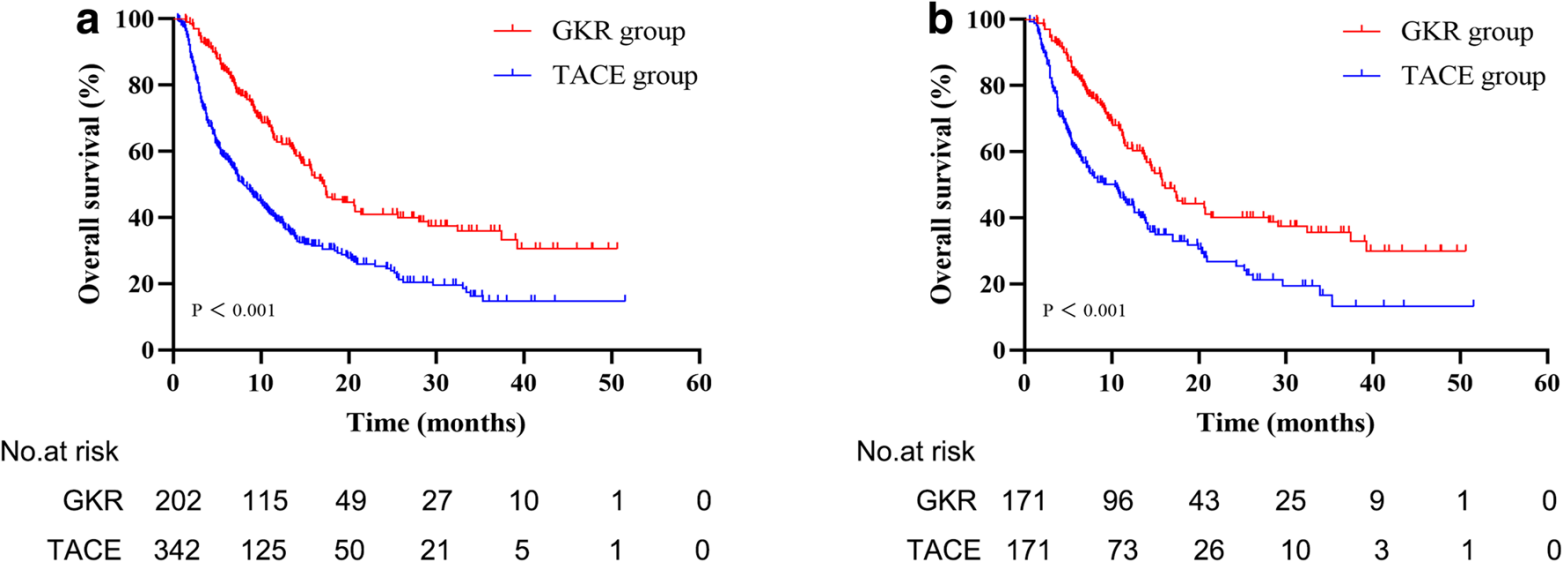
**A** GKR had significantly improved overall survival compared to TACE before propensity score matching (17.2 vs. 8.0 months). **B** GKR had significantly improved overall survival compared to TACE in the matched cohort (15.8 vs. 10.4 months). Abbreviations: *GKR* gamma knife radiosurgery, *TACE* transcatheter arterial chemoembolization

**Fig. 2:**
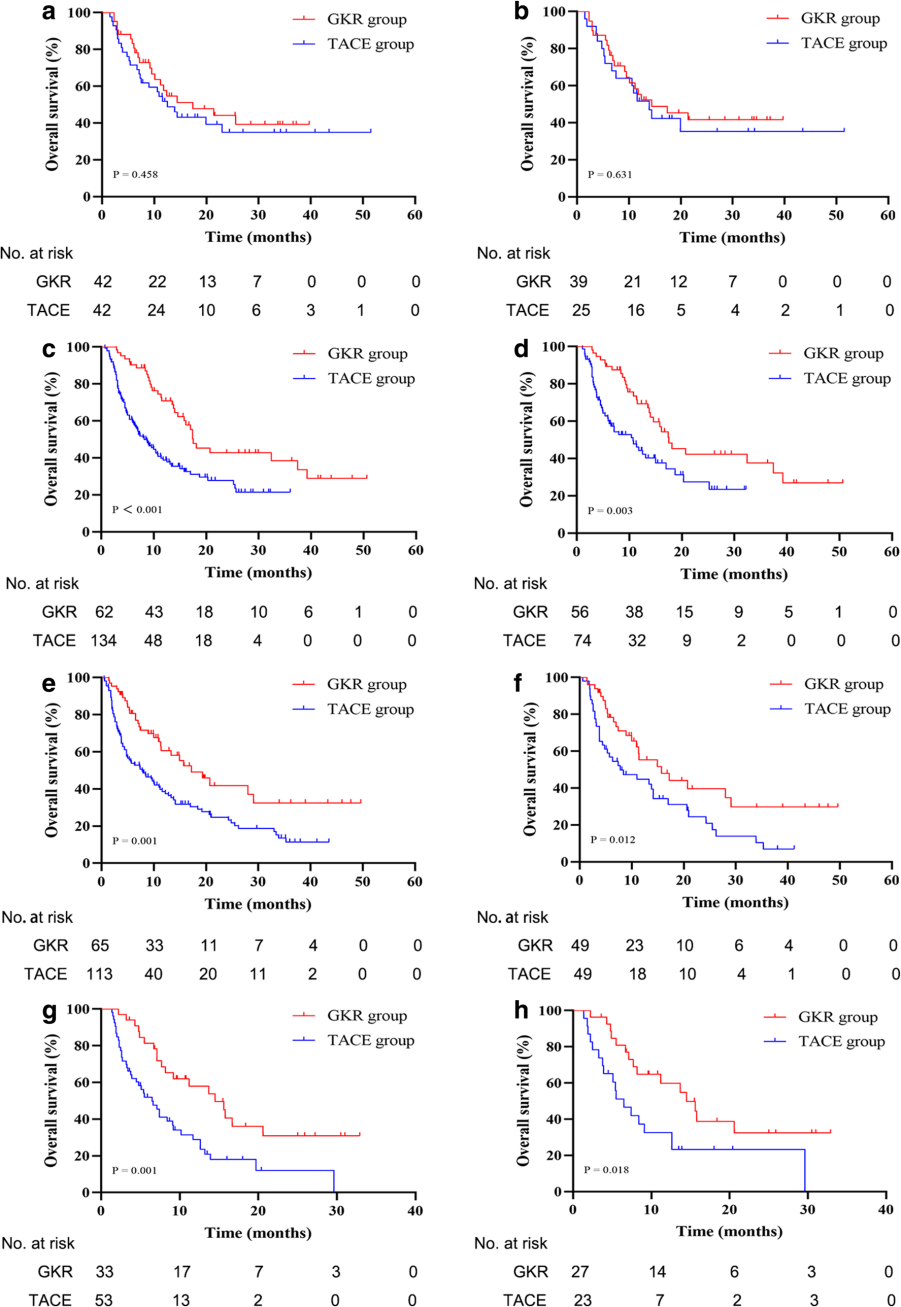
Overall survival curves for the patients with portal vein tumor thrombus (PVTT) type I (**A**), II (**C**), III (**E**), and IV (**G**) who received GKR and TACE are shown before propensity score matching. Overall survival curves for the patients with PVTT type I (**B**), II (**D**), III (**F**), and IV (**H**) who received GKR and TACE are shown after propensity score matching. Abbreviations: *GKR* gamma knife radiosurgery, *TACE* transcatheter arterial chemoembolization

At the cutoff date (September 1, 2021), in the pooled cohort, 102 (50.5%) patients in the GKR group and 239 (69.9%) patients in the TACE group had died. The mOS was 17.2 months (14.4–20.0) in the GKR group and 8.0 months (6.4–9.6) in the TACE group (*p* < 0.001, Fig. 1A). The cumulative OS rates at 12, 18, and 24 months were 62.7, 45.5, and 40.3% in the GKR group, and 38.7, 30.0, and 24.8% in the TACE group. The OS rates were significantly higher in patients in the GKR group than in those in the TACE group (*p* < 0.001). In subgroup analysis according to the type of PVTT, patients in the GKR group had a significantly longer OS than those patients with PVTT type II, III and IV in the TACE group (17.5 vs. 8.7 months, *p* < 0.001, Fig. 2C; 17.2 vs. 7.8 months, *p* = 0.001, Fig. 2E; 14.5 vs. 6.5 months, *p* = 0.001, Fig. 2G; respectively). However, there was no significant difference in the OS between the two groups of patients with PVTT type I (17.4 vs. 12.5 months, *p* = 0.458, Fig. 2A).

In the matched cohort, 88 (51.5%) patients in the GKR group patients and 117 (68.4%) in the TACE group patients died. The GKR group showed a mOS of 15.8 (13.0–18.6) months, which was higher than that of 10.4 (7.2–13.6) months in the TACE group (*p* < 0.001, Fig. 1B). The cumulative OS rates at 12, 18, and 24 months were 60.8, 44.4, and 39.6 in the GKR group, and 43.7%, 32.1%, and 25.5% in the TACE group (*p* < 0.001), respectively. Patients with PVTT type II, III and IV in the GKR group had also a significantly longer OS than their counterparts the TACE group (17.5 vs. 10.7 months, *p* = 0.003, Fig. 2D; 15.7 vs. 7.8 months, *p* = 0.012, Fig. 2F; 14.5 vs. 6.5 months, *p* = 0.018, Fig. 2H; respectively). There was no significant difference in the OS between the two groups of patients with PVTT type I (14.4 vs. 13.9 months, *p* = 0.631, Fig. 2B).

### Exploratory subgroup analysis of associated factors in the matched cohorts

In exploratory subgroup analyses of OS after PSM (Supplementary Fig. 2), the forrest-plot suggested lack of benefit of GKR over TACE in patients with PVTT type I and tumor size < 5 cm.

### Factors associated with overall survival in the pooled and matched cohorts

We performed univariate and multivariate Cox regression analyses to confim prognostic independent factors. Before PSM, GKR, number of tumors < 2, tumor diameter < 5 cm, and ALP < 125 U/L were confirmed as independent positive prognostic factors for OS (all *p* < 0.005, Supplementary Table 1). After PSM, GKR, tumor diameter < 5 cm, and ALP < 125 U/L were all confirmed as independent positive prognostic factors for OS (all *p* < 0.005, Table 2).

**Table 2:** Univariate and multivariate Cox regression analysis of overall survival after PSM

Variable	Univariable Cox regression				Multivariable Cox regression		
	HR	95%CI	*p*		HR	95%CI	*p*
Sex (male/female)	1.119	0.706–1.776	0.632				
Age (≥60/<60 years)	0.780	0.581–1.047	0.098				
Child–Pugh class (B/A)	1.604	1.199–2.147	0.001		1.323	0.98–1.787	0.068
Number of tumor (≥2/ <2)	1.604	1.119–2.302	0.010		1.343	0.934–1.930	0.112
Tumor diameter (≥5/<5 cm)	2.187	1.461–3.272	<0.001		1.778	1.180–2.679	0.006
AFP (≥400/<400 ng/ml)	1.281	0.972–1.686	0.078				
ALP (≥125/<125 U/L)	2.123	1.539–2.928	<0.001		1.847	1.315–2.594	<0.001
Platelet (<100,000/ ≥100,000/μL)	1.376	0.999–1.896	0.051				
ALT (≥ 40/< 40U/L)	1.220	0.920–1.617	0.167				
Leukocyte (<4000/≥4000/μL)	1.278	0.898–1.818	0.173				
Cheng’s type of PVTT			0.227				
I	1.000						
II	0.652	0.404–1.050	0.078				
III	0.769	0.510–1.158	0.208				
IV	0.925	0.605–1.413	0.717				
HBV (positive/negative)	1.140	0.854–1.522	0.375				
HCV (positive/negative)	1.553	0.730–3.302	0.253				
Alcoholism (positive/negative)	1.082	0.819–1.428	0.581				
Lymph node metastasis (yes/no)	1.196	0.907–1.577	0.205				
Extrahepatic metastases (yes/no)	1.084	0.805–1.459	0.595				
Previous therapy (yes/no)	0.885	0.668–1.172	0.393				
Treatment (GKR/TACE)	0.553	0.419–0.731	<0.001		0.563	0.426–0.744	<0.001

*PSM* propensity score matching, *HR* hazard ratio, *PVTT* portal vein tumor thrombus, *AFP* alpha fetoprotein, *ALP* alkaline phosphatase, *ALT* alanine transaminase, *HBV* hepatitis B virus, *HCV* hepatitis C virus, *GKR* gamma knife radiosurgery, *TACE* transcatheter arterial chemoembolization

## Discussion

To the best of our knowledge, this is the first study to investigate the efficacy of GKR vs. TACE monotherapy in the treatment of HCC-PVTT patients. Treatment options are limited for HCC patients with PVTT [17]. The Barcelona Clinic Liver Cancer staging system classified patients with PVTT to be at an advanced stage and recommended atezolizumab plus bevacizumab as the first-line treatment [5]. However, there is still controversy regarding the best locoregional treatment for HCC patients with PVTT [9].

In this study, the TACE group had heavier tumor burden and higher AFP levels than the GKR group before PSM. Therefore,we performed PSM to reduce bias due to confounding in baseline characteristics between the two groups. We found that GKR was superior to TACE with respect to the survival time for patients with PVTT II, III, and IV (all *p* < 0.05). However, no significant difference in the OS before and after PSM was observed for patients with PVTT I. These results have implications for the treatment of HCC patients with PVTT.

Although both GKR and TACE are local therapies to induce tumor cell death, the two modalities differ substantially in their principles and processes of treatment, which might be underly differences in treatment efficacy in HCC. A growing number of studies reported that TACE can be safely conducted in patients with incomplete obstruction of the main portal vein or formation of abundant compensatory collateral branches of the portal vein, or recanalized portal vein by portal vein stenting despite complete obstruction [18–21]. However, in cases of complete obstruction of the main portal vein by tumor thrombi with few collateral branches formed, TACE is contraindicated [18]. Patients with PVTT type II, III, and IV often experience severe obstruction of the portal vein, which might be associated with the poor survival time of these patients in the TACE. In contrast, GKR, an external RT method, is the most widely used form of stereotactic radiosurgery in America due to its precision and the efficiency of radiation delivered in a single session [22, 23]. With development of RT technology, radiation dosage of targeted regions can be increased while achieving protection of the adjacent healthy tissues, thereby making it suitable for HCC patients with all types of PVTT [9, 24, 25]. In addition to sustaining local control with low toxicity, local RT also induces immunogenic cell death, increase the release of tumor antigens and activate the immune system [12, 26–28].

A retrospective study reported that HCC patients with PVTT who received GKR plus TACE had longer OS than those who received TACE alone (9.7 vs. 6.2 months, respectively; *p* < 0.001) [13]. A study by Li and colleagues reported that mOS values of 8 vs. 10 months in the intensity-modulated radiation therapy (IMRT) and SBRT groups (*p* = 0.165) [29]. In addition, Silva et al. conducted a meta-analysis and reported that the mOS of HCC patients who received TACE was 8 months [21]. In a phase II study, the mOS of HCC patients with PVTT receiving selective internal radiation (SIRT) was 13 months [30]. In our study, patients in the GKR group experienced longer survival time than those in the TACE after PSM (15.8 vs. 10.4 months, *p* < 0.001). Additionally, it is important to note that the total cost of GKR and TACE alone is similar in China. Due to non-invasive nature of GKR, patients are more willing to accept the GKR rather than TACE. In summary, taking into consideration overall efficacy and costs, GKR appears to be a more feasible therapeutic option for HCC patients with PVTT.

Next, we explored the prognostic factors for HCC patients with PVTT and found that GKR was independently associated with long‐term OS. In addition, the tumor diameter ≥ 5 cm and ALP ≥ 125 U/L were associated with inferior OS after PSM. These findings are in line with previous reports linking these factors with poor prognosis in HCC patients [31–33].

A seemingly unexplainable phenomenon was observed in our study. Previous studies showed that HCC patients with PVTT type I had longer mOS than those with PVTT type II-IV [34, 35]. However, after PSM, the outcomes of PVTT type I in the GKR group did not appear to be better than those of other patients. The poor OS in patients with PVTT type I might be an artifact resulting from the small sample size of PVTT type I (*n* = 39).

This study has several limitations. Firstly, this was a retrospective study and as such it is prone to potential bias. We conducted PSM to avoid selection bias, but we cannot exclude potential confounding factors. Secondly, the information on the adverse effects was limited. Finally, the sample size of the GKR group was relatively small, although our study had the largest HCC patients with PVTT ever reported to our knowledge.

## Conclusions

In conclusion, in this multicenter retrospective study, GKR showed better OS than TACE in HCC patients with PVTT, especially in those with PVTT type II, III and IV. These findings have significant implications for the treatment of HCC patients with PVTT. Prospective randomized trials are needed to demonstrate the potential benefit and safety of GKR for patients with HCC.

## Supplementary Information

Below is the link to the electronic supplementary material.

Supplementary file1 (DOCX 517 KB)

## Data Availability

All data generated or analyzed during this study are included in this article and its supplementary material files. Further enquiries can be directed to the corresponding author (Lanpaoxiansheng@126.com).

## Declarations

### Animal Research

This research did not involve animal experiments.
