# Supplementary material for: Gamma knife radiosurgery versus transcatheter arterial chemoembolization for hepatocellular carcinoma with portal vein tumor thrombus: a propensity score matching study
Source: Hepatol Int. 2022 Jun 21;16(4):858–67. doi: 10.1007/s12072-022-10339-2 (PMC9349123; doi:10.1007/s12072-022-10339-2)

| **Supplementary Table 1. Univariate and multivariate Cox regression analysis of overall survival before PSM** | | | | | | | |
| --- | --- | --- | --- | --- | --- | --- | --- |
|  | Univariate Cox regression | | |  | Multivariate Cox regression | | |
| Variable | HR | 95%CI | P |  | HR | 95%CI | P |
| Sex (male/female) | 1.020 | 0.731-1.424 | 0.907 |  |  |  |  |
| Age (≥60/<60 years) | 0.750 | 0.594-0.947 | 0.016 |  | 0.926 | 0.725-1.182 | 0.535 |
| Child-Pugh class (B/A) | 1.447 | 1.151-1.819 | 0.002 |  | 1.221 | 0.959-1.554 | 0.105 |
| Number of tumor (≥2/<2) | 1.825 | 1.360-2.448 | < 0.001 |  | 1.521 | 1.125-2.056 | 0.006 |
| Tumor diameter (≥5/<5 cm) | 1.839 | 1.374-2.461 | < 0.001 |  | 1.493 | 1.106-2.014 | 0.009 |
| AFP (≥400/<400 ng/ml) | 1.351 | 1.088-1.677 | 0.006 |  | 1.177 | 0.941-1.473 | 0.154 |
| ALP (≥125/<125 U/L) | 1.851 | 1.442-2.375 | < 0.001 |  | 1.504 | 1.142-1.980 | 0.004 |
| Platelet (<100000/≥100000/μL) | 1.318 | 1.026-1.692 | 0.031 |  | 1.253 | 0.971-1.619 | 0.083 |
| ALT (≥40/<40U/L) | 1.347 | 1.078-1.683 | 0.009 |  | 1.179 | 0.926-1.502 | 0.182 |
| Leukocyte (<4000/≥4000/μL) | 1.308 | 0.995-1.720 | 0.054 |  |  |  |  |
| Cheng’s type of PVTT |  |  | 0.022 |  |  |  | 0.055 |
| I | 1.000 |  |  |  | 1.000 |  |  |
| II | 0.561 | 0.381-0.825 | 0.003 |  | 0.609 | 0.411-0.901 | 0.013 |
| III | 0.728 | 0.534-0.993 | 0.045 |  | 0.689 | 0.503-0.942 | 0.020 |
| IV | 0.828 | 0.605-1.133 | 0.239 |  | 0.749 | 0.545-1.03 | 0.075 |
| HBV (positive/negative) | 1.013 | 0.813-1.263 | 0.908 |  |  |  |  |
| HCV (positive/negative) | 1.307 | 0.716-2.383 | 0.383 |  |  |  |  |
| Alcoholism (positive/negative) | 1.035 | 0.834-1.284 | 0.757 |  |  |  |  |
| Lymph node metastasis (yes/no) | 1.273 | 1.026-1.581 | 0.029 |  | 1.117 | 0.892-1.399 | 0.336 |
| Extrahepatic metastases (yes/no) | 1.109 | 0.877-1.402 | 0.389 |  |  |  |  |
| Previous therapy (yes/no) | 0.693 | 0.546-0.88 | 0.003 |  | 1.065 | 0.82-1.382 | 0.637 |
| Treatment (GKR/TACE) | 0.504 | 0.399-0.636 | < 0.001 |  | 0.536 | 0.418-0.687 | < 0.001 |
| Abbreviations: PSM, propensity score matching; HR, hazard ratio; PVTT, portal vein tumor thrombus; AFP, alpha fetoprotein; ALP, alkaline phosphatase; ALT, alanine transaminase; HBV, hepatitis B virus; HCV, hepatitis C virus; GKR, gamma knife radiosurgery; TACE, transcatheter arterial chemoembolization. | | | | | | | |

**Supplementary Fig. 1.** Patient selection flow chart. Abbreviations: GKR, gamma knife radiosurgery; TACE, transcatheter arterial chemoembolization; HCC, hepatocellular carcinoma; ECOG, Eastern Cooperative Oncology Group performance status; PVTT, portal vein tumor thrombus.


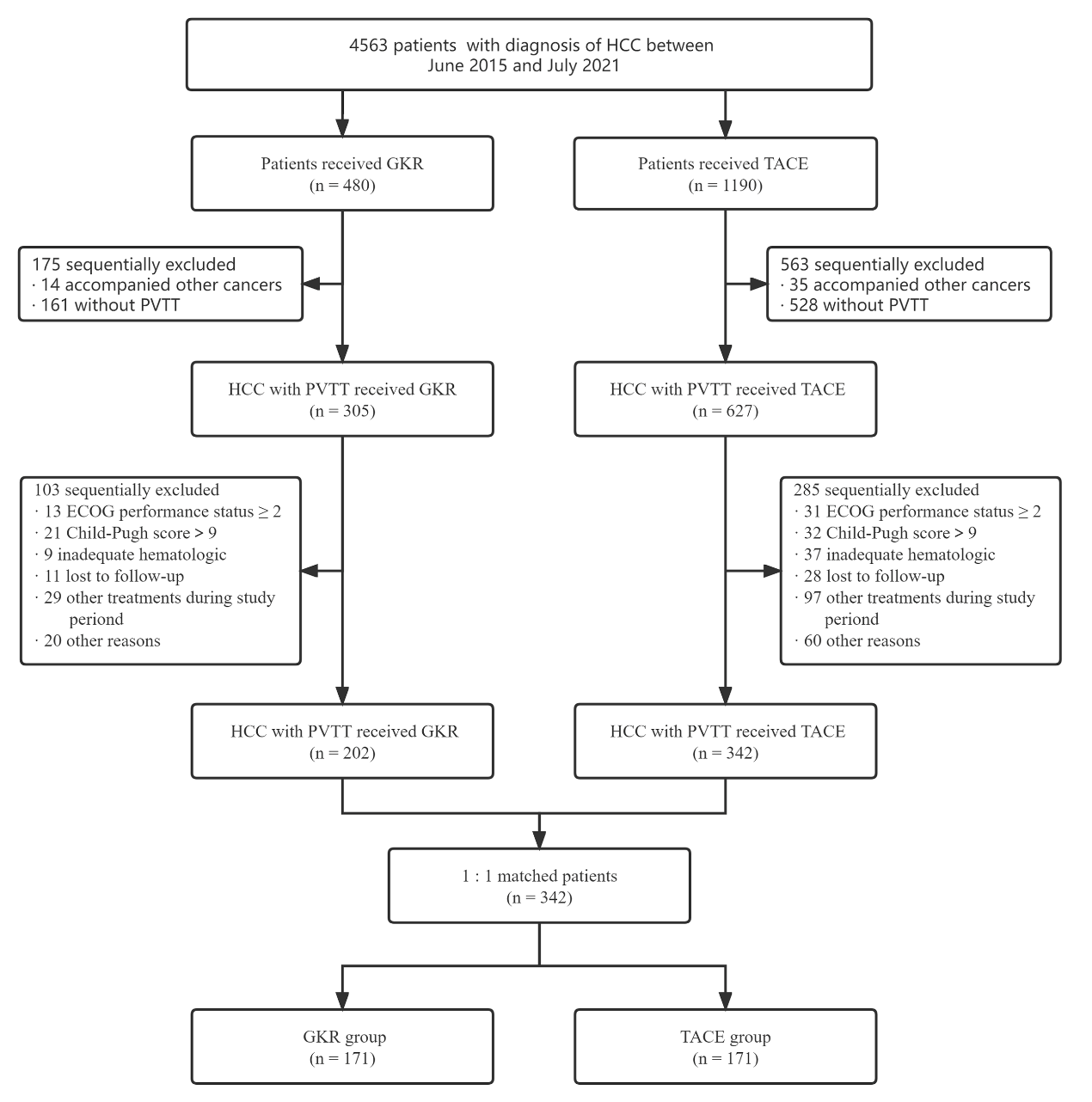


**Supplementary Fig. 2**: Exploratory subgroup analysis of associated factors. Forest plots show factors associated with overall survival. Abbreviations: HR, hazard ratio; AFP, alpha fetoprotein; ALP, alkaline phosphatase; ALT, alanine transaminase; PVTT, portal vein tumor thrombus; HBV, hepatitis B virus; GKR, gamma knife radiosurgery; TACE, transcatheter arterial chemoembolization.


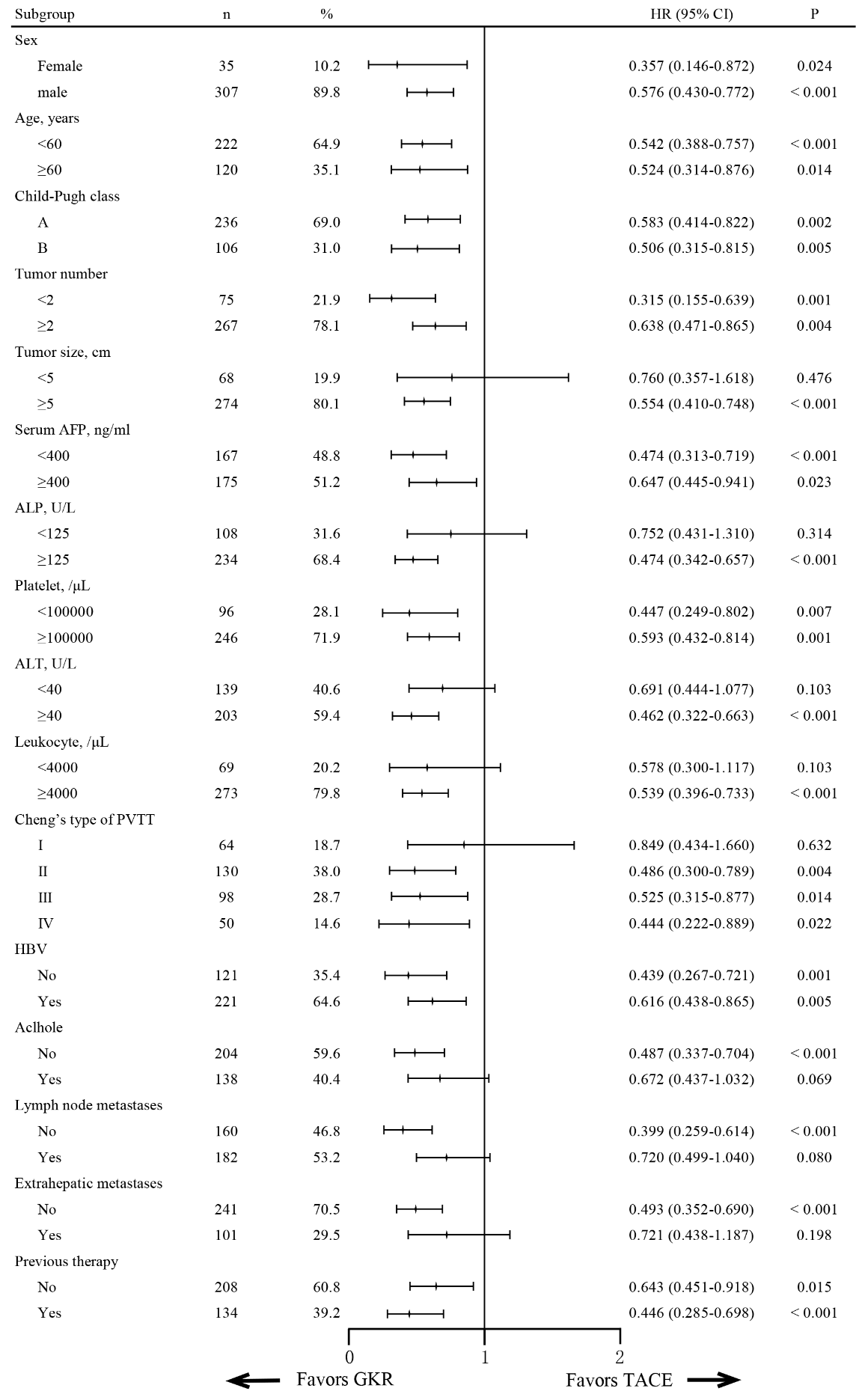

Supplement: Supplementary file 1 — Supplementary file1 (DOCX 517 KB) [file 12072_2022_10339_MOESM1_ESM.docx]
